# Coproducing a culturally sensitive storytelling video intervention to improve psychosocial well-being: a multimethods participatory study with Nepalese migrant workers

**DOI:** 10.1136/bmjopen-2024-086280

**Published:** 2025-02-17

**Authors:** Priyamvada Paudyal, Sharada Prasad Wasti, Pimala Neupane, Jib Lal Sapkota, Carol Watts, Kavian Kulasabanathan, Ram Silwal, Anjum Memon, Prajaya Shukla, Ram Sharan Pathak, Daniel Michelson, Clio Berry, Alice Moult, Padam Simkhada, Edwin R van Teijlingen, Jackie Cassell

**Affiliations:** 1 Institue for Global Health and Wellbeing, School of Medicine, Keele University, Newcastle-under-Lyme, UK; 2 Department of Primary Care and Public Health, Brighton and Sussex Medical School, University of Sussex, Brighton, UK; 3 School of Human Sciences, Faculty of Education, Health and Human Sciences, University of Greenwich, London, UK; 4 Central Department of English, Tribhuvan University, Kirtipur, Nepal; 5 School of Media, Arts and Humanities, University of Sussex, Brighton, UK; 6 Wolfson Institute of Population Health, Queen Mary University London, London, UK; 7 Green Tara Nepal, Kathmandu, Nepal; 8 Nepal Khabar, Kathmandu, Nepal; 9 Central Department of Population Studies, Tribhuvan University, Kirtipur, Nepal; 10 Department of Child and Adolescent Psychiatry, King’s College London, London, UK; 11 Impact Accelerator Unit, School of Medicine, Keele University, Newcastle-under-Lyme, UK; 12 School of Human and Health Sciences, University of Huddersfield, Huddersfield, UK; 13 Faculty of Health and Social Sciences, Bournemouth University, Bournemouth, UK; 14 Centre for Health Service Studies, University of Kent, Canterbury, UK

**Keywords:** Health, Community-Based Participatory Research, Nepal

## Abstract

**Abstract:**

**Objective:**

This study aimed to coproduce a culturally adaptive storytelling video intervention to support the psychosocial well-being of Nepalese migrant workers.

**Design:**

A multimethods participatory study was conducted involving three different but interconnected phases: (1) formative research involving a systematic review, pilot survey and stakeholder consultations; (2) exploration and analysis of Nepalese literature relevant to contemporary migration; and (3) coproduction of a storytelling video intervention, using participatory workshops.

**Participants and settings:**

Convenience sample of outgoing and returnee migrant workers from the Gulf Cooperation Council (GCC) countries, their families and other relevant stakeholders in Dhading District of Bagmati Province, Nepal.

**Results:**

The systematic review of 33 included studies identified five key health issues: mental health; occupational hazards; sexual health; healthcare access; and infectious diseases. In the survey (n=60), workers reported various health problems including fever/common cold (42%); mental health problems (25%); and verbal abuse (35%). Twenty interviewees identified issues related to physical health (eg, pneumonia, kidney disease) as well as mental health (eg, anxiety, depression). Nepalese literary resources primarily portrayed themes of: separation; hopelessness and helplessness; and poor workplace environments. Drawing on these findings and iterative workshops with stakeholders, a culturally sensitive storytelling video intervention was coproduced to support the psychosocial well-being of Nepalese migrant workers in GCC countries. The intervention used an animated video format with audio narration and subtitles, presenting a story centred around the struggles of an archetypal male migrant worker and their use of coping strategies for dealing with adversities.

**Conclusions:**

This is a feasibility study conducted in a single district of Nepal; as such, the findings should be generalised cautiously. Despite these limitations, the project is testament to the value of participatory methods in the development of culturally sensitive public health interventions for marginalised groups, and points to the utility of coproduced storytelling formats in migrant health contexts. Future research is needed to evaluate feasibility and acceptability of the intervention as well as the outcomes and experiences of migrant workers who engaged with the video.

**STRENGTHS AND LIMITATIONS OF THIS STUDY**

- The study was guided by participatory research principles and involved migrant workers, their families and stakeholders from civil society (including advocacy organisations), government and labour recruitment agencies.
- The study team was multidisciplinary and had expertise in public health, psychology, demography, sociology, media and arts and humanities.
- While the study was conducted in one district of Nepal with a small number of migrant workers, the demographics of participants were similar to the wider population of Nepalese migrant workers.
- It was not possible to gather formal feedback on the storytelling video due to COVID-19 restrictions imposed towards the end of the study.

## Introduction

 Labour migration contributes significantly to the sociocultural and economic development of both home and host countries; developed nations benefit through workforce growth, productivity and innovation, while remittances from migrant workers provide a crucial source of income for many low-income and middle-income countries.^1^ However, migrant workers face a range of vulnerabilities while working abroad and are at high risk of poor health outcomes.^2 3^ In particular, workers from low- and middle-income countries are over-represented in high-risk occupations and are more likely to face unfavourable social conditions such as precarious employment and poor living conditions.^4 5^ Targeted public health interventions are needed to address these occupational and psychosocial risks, using delivery formats that can overcome the limited access to social protection and associated linguistic and cultural barriers faced by these groups.^6^

Nepal is an Official Development Assistance country that includes a large diaspora of workers serving abroad. Almost half of all households have at least one family member who is currently working or has previously worked abroad.^7^ Poverty, limited employment opportunities, and deteriorating agricultural productivity in the country has resulted in increased international labour migration.^8^ Approximately 2.1 million Nepalese (>7% of the total population) work abroad (excluding India), sending around US$8 billion home every year, and remittance contributes to one-quarter of the nation’s gross domestic product.^9^ Malaysia and the six countries of the Gulf Cooperation Council (GCC) (Saudi Arabia, Kuwait, Bahrain, Oman, Qatar, and UAE (United Arab Emirates)) are the most common destinations, receiving 85% of Nepal’s outward labour force.^9^

International perceptions of Nepalese migrants have shifted from ‘global warriors to global workers’.^10^ Nepalese workers, particularly in GCC countries, are often employed in ‘difficult, dirty, and dangerous’ occupations known as ‘3D’ jobs.^10^ Prolonged working hours (>60 hours per week) are commonplace, often involving heavy manual labour and extreme heat. Such working conditions can result in occupational injuries, while risky behaviours outside of the workplace (eg, sexual risk-taking and substance use) compound poor health outcomes. Furthermore, male workers often leave partners and young children behind in socially and economically vulnerable environments. Intersecting health risks are faced by female migrants, who are mostly engaged in domestic work in the GCC countries and vulnerable to forced labour, abuse and sex trafficking.^9^,^11^

The relationship between migration and storytelling is ancient. Migrants from across the globe have depicted the challenges of departure, travel and arrival through stories, poems and songs. Storytelling can be a measure of home, a form of refuge, and a means of adaptation across borders. Given the centrality of personal and communal narratives in lay representations of risk and resilience, stories have been increasingly used to disseminate public health messages.^12^,^14^ Drawing on concepts from communication theory^15^ and social cognitive theory,^16^ mechanistic descriptions of storytelling interventions have highlighted key processes including transportation (reducing cognitive resistance by encouraging the reader/listener to enter into the world of the narrator); and identification (enhancing the listener/viewer’s acceptance of the values and beliefs portrayed in the story through narrative persuasion and modelling).^17^ Evidence shows that storytelling can effectively engage disadvantaged populations and lead to changes in health-related knowledge, attitudes, beliefs and behaviours across a wide variety of health conditions, including depression, cardiovascular disease, sexual health problems and cancer.^18^,^21^ The potential benefits of storytelling approaches have also been demonstrated in recent research on health messaging during the COVID-19 pandemic.^22^

Migrants’ narratives are often constrained and reinterpreted according to institutional needs and expectations and are likely to be influenced by ‘invisible power asymmetries’.^23^ A recent study exploring the visual portrayal of migrants suggests that the media predominantly portrays migrants as negative, often living in poverty, and being a risk for the destination country,^24^ and the sending country.^25^ Migrants lack platforms to use their voice, share their own stories and participate in storytelling activities. Coproduction is widely promoted as a method for sharing power and delivering mutual benefits to stakeholders.^26^ Power dynamics are influenced by who has authority over resources and whose knowledge is valued within that context. While telling, sharing and cocreating stories with migrants, it is important to draw on their individual and collective experiences and avoid reinforcing harmful and fear-based narratives of portraying migrants as victims or reduce them to just their ‘migrantness’.^27^ Providing opportunities for migrants to share their authentic, whole and multidimensional narratives can empower migrant communities.^27^ Hence, our interdisciplinary study aimed to coproduce a culturally adaptive storytelling intervention to support the psychosocial well-being of migrant workers, focusing on outgoing and returning Nepalese migrant workers working in GCC countries.

## Methods

The participatory design involved coproduction and prototyping across three interconnected phases,^28^ in line with recommendations on coproduction from the UK’s National Institute for Health and Care Research^29^ and taking a comprehensive approach consistent with the World Health Organisation’s technical guidance on health promotion for migrants.^30^ This study is part of a larger project. Findings from the systematic review and mixed-method study that informed this coproduction process have already been published^4 31^; therefore, this paper only provides a brief summary of their methodology and findings to maintain conciseness.

### Phase 1: exploring health and well-being issues of Nepalese migrant workers

A preregistered systematic review explored the health and well-being of Nepalese migrant workers in GCC countries and Malaysia. Studies were eligible if they included: (1) Nepalese migrant workers aged 18 years or older working in, or returning from, the GCC countries or Malaysia; (2) primary studies into health and well-being status/issues; and (3) were published in English. Full results of this systematic review have been published elsewhere.^4^

In addition, a mixed-method study was conducted to better understand health priorities and existing health service provision for Nepalese migrants in GCC countries. The study was conducted in the Dhading district of Bagmati Province, Nepal, which was selected for its high number of migrant workers. A face-to-face survey was conducted with 60 adult returnee migrants from GCC countries, and 12 of them completed individual qualitative interviews. An additional eight qualitative interviews were completed with family members of migrant workers, and stakeholders from non-governmental organisations (NGOs), government, advocacy organisations and recruitment agencies. The survey and the interviews covered a wide range of health and well-being issues including workplace abuse, information on predeparture medical exams and trainings, and healthcare access in destination countries. Details of this mixed-method study have been published elsewhere.^31^

### Phase 2: identification, exploration and analysis of relevant literary resources

We searched for literary resources published in Nepali which portrayed the health and well-being of Nepalese migrant workers and their families. To understand the wider cultural contexts of migration, the resources were not limited to migration to GCC countries. The search strategy involved four steps. First, we liaised with the Arts and Humanities Departments of major academic institutions such as the Central Department of English, Kirtipur and Bhaktapur Multiple Campus, Bhaktapur in Nepal to enquire about relevant resources. Second, we approached contemporary writers (with publications in the migration field), professors of literature (Nepali and English) based in Kathmandu, using a snowballing approach.^32^ Third, we visited key libraries in Kathmandu such as Central Library, Kirtipur and Madan Pustakalya. Fourth, we consulted with book publishers and book stores in Kathmandu such as Ratna Pustak Bhandar and Sajha Publication. Poems, stories, novels, autobiographical accounts, travelogues and songs (both published and recorded) were reviewed. Once the relevant resources were identified from these multiple channels, we analysed the texts and identified key themes using a narrative analysis approach.^33^ The analysis focused not only on story content, but also considered how the stories were told and their social contexts.^33^

### Phase 3: coproduction of the storytelling intervention

Building on the evidence from the preceding phases, we iteratively coproduced the storytelling intervention by engaging with migrant workers and their families. The collaboration ensured that the intervention was attuned to the needs and aspirations of migrant workers. Returnee migrants and their families were encouraged to engage throughout the process according to their needs and availability. The process aimed to dissolve hierarchies and promoted respect by providing equal opportunities to contribute to the decision-making processes.^34 35^

A coproduction group (n=12) was formed with six migrant workers, three family members, two researchers (SPW and PN) and a literary scholar (JLS). The researchers and the literary scholar were trained on the theory and practicalities of the coproduction process.^28 36 37^ Sufficient time was allowed before the coproduction process to encourage active involvement of migrant workers and their families (eg, arranging informal meetings, visiting and interacting in community settings). The group was provided with information on various processes involved in coproduction, how their engagement and input benefit the process, expected contributions from them, group dynamics and respecting diverse perspectives, the anonymity and confidentiality of the discussion and the voluntary nature of their contribution. Three coproduction workshops were held (facilitated by SPW and JLS and close observation by PN) to reflect on the findings from phases 1 and 2, and to discuss the nature and the content of the central story.

### Patient and public involvement

This study involved migrant workers, their families and other stakeholders throughout the lifespan of the project.

## Results

**Figure 1:**
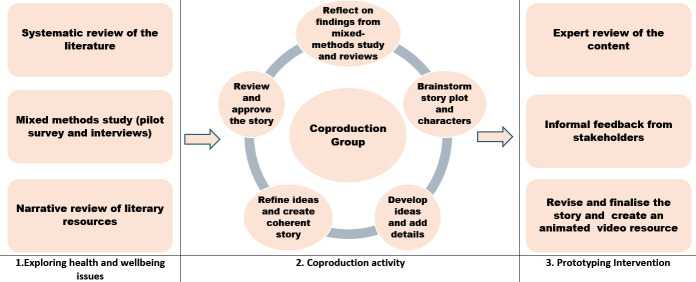
Framework for coproduction of the storytelling intervention (adapted from Hawkins *et al*^28^).

The activities conducted for the coproduction of the storytelling intervention are summarised in figure 1; the overall process took 12 months.

### Phase 1: exploring health and well-being issues of Nepalese migrant workers

The systematic review identified 33 eligible studies for inclusion. Twelve of these studies were conducted in Qatar, eight in Malaysia, nine in Nepal, two in Saudi Arabia and one each in UAE and Kuwait. Five key health and well-being-related issues were identified from the review: mental health; occupational hazards; sexual health; healthcare access; and infectious diseases.^4^ The review highlighted the need for improved health support for migrant workers, along with stronger legislation around acceptable working conditions and appropriate predeparture training. The findings suggested an urgent need for progressive policy changes, both in Nepal and destination countries, to better safeguard the health of labour migrants and improve their access to essential healthcare service.^4^

Sixty returnee migrants (58 males/2 females) took part in the survey. They reported suffering from various physical and mental health issues during their stay in GCC countries including fevers and other common cold/influenza symptoms (42%); mental health problems (25%); and verbal abuse (35%). In the qualitative interviews, 20 participants took part: 10 returnee migrants, four family members and six key stakeholders working in organisations related to international migration. Participants reported physical health problems (eg, pneumonia, dehydration, kidney disease) as well as mental health issues (eg, anxiety, loneliness, depression). The workers raised concerns about the utility and appropriateness of predeparture training, and the authenticity of medical tests and reports in Nepal. Language difficulties, alongside issues related to payment, insurance and support at work, were cited as barriers to accessing healthcare in destination countries.^31^ The study highlighted an urgent strategic need for enforcing compulsory predeparture orientation and appropriate medical screening in Nepal, as well as fair labour practices and full health insurance coverage in GCC countries. Also, the study emphasised the need for a greater collaboration between the government of Nepal and GCC countries to ensure necessary regulatory frameworks are in place for safeguarding the health and well-being of Nepalese migrant workers.^31^

### Phase 2: identification, exploration and analysis of relevant literary resources

A total of 167 literary resources were identified during the search, and 49 were eligible for inclusion (table 1), most were written by male authors. Earlier literary pieces, published before 2010, focused on migration to join either the Indian or British army. More contemporary work (post 2011) centred around the pain, penury and predicament of migrant workers in Gulf states and Malaysia. A few literary resources described positive migration stories such as achieving justice by punishing the trafficker, saving the life of the victim, and gaining economic independence through entrepreneurship. The texts portrayed separation (death and loss, mutual unhappiness and longing); hopelessness and helplessness (isolation, poverty, uncertainty, powerlessness and worthlessness); and poor workplace environments (extreme temperatures and workplace injuries) (table 2). The narrative resources were key in framing how the story was told by ‘Suman,’ who is the protagonist in the story (box 1). Their influence shaped the narrative tone used to describe the experience of adversity during the period of foreign employment, the desired behaviour changes through the course of the narrative and the formulation of messages in a positive and empowering way.

**Table 1:** Types of literature identified

Literary resources	Items reviewed (n)	Eligible items (n)
Songs	61	22
Novel	40	17
Poems	26	6
Stories	31	3
Biographies/travelogue	9	1
Total	167	49

**Table 2:** Themes, subthemes and example scripts form the literature

Key themes	Subthemes	Example scripts
Separation	Death and loss	They fly up into the sky, but return in a coffin,What a bad omen!…The vermilion pot fell on the ground here,And a life was lost there,No one to offer [them] water in that unknown landThe uncertainty of migrant life…Alas! The only hope of returning home is lost.(Saput, 2018)
	Mutual unhappiness	The husband left for foreign lands with tears rolling down,Leaving me behind crying on my own.My dear [husband] entered foreign lands,But he has neither money nor happiness.Instead, I heard that he is there crying…Why are you earning, my dear,Leaving me here with my desires unfulfilled.(Dashaudi, 2017)
	Longing	Who will understand the pain of migrants,No certainty in life or death,My mother, I cry reminiscing about my village…No certainty in life or death, my son, return home.(Pariyar, 2016)
Hopelessness and helplessness	Poverty	The money lenders come to harass me whilst I’m in bed,Harass me whilst I’m in bedI am frightened every momentI left home in a hurry to earnTake care of yourself in the village [my beloved wife]Came to this foreign land, Saudi, Qatar(Thapa and Shyantan, 2014)
	Isolation	People are not as friendly here as in my village…The only friend I have is my own shadow,Nothing is certain in this foreign lifeLiving in foreign land with the hope to return homeThis heart is always heavy.(KC, 2019)
	Uncertainty	I was in misfortune. I was not recovered fully. Where to go at that moment? There is no alternative about what to do and not to do when we are at the moment of misfortune and suffering in the foreign land. No one asks with a single word saying what did you eat, how are you? When we die, we become worthless like the death of dog or cat. When I remember that situation, tears still roll down from my eyes.(Bangdel, 1948)
	Powerlessness	He lured women whose husbands were abroad. Several women who became his victims were like this… they would traffic them, alluring them with marriage, job, or foreign employment.(Bhandari, 2013)
	Worthlessness	They do not know anything [in the foreign land] and then happen to be like a crow in the fog… who cares about an unknown person?(Adhikari, 2011)
Poor workplace environments	Extreme temperatures	You used to tell me not to go out in the cold,But, mother, I now work in minus 10 degrees,I’ve just come to realise why people hang themselves.This is the way of foreign life,No matter how hard I work, it’s never enough.(Kharal and Kharal, 2018)
	Workplace injuries	‘Heard that Soome works above the 50th floor. What happens if he falls?’‘Shankare’s hand was completely cut off by machine.’(Bhandari, 2013)

Box 1 **Story script**

**Gulfemployment and Suman’shealthylifestyle**

*My name is Suman.*

*I live in Thakre, a small village in Dhading District, north-west of Kathmandu. Perhaps you know it? I’m 24 and come from an ordinary family—I’m the eldest child. I’ve always worked hard, helping out with chores, and supporting my parents by taking on farm jobs during the vacations. I really liked studying and did well at school—I passed my exams on the first attempt from Shree Mahakali Higher Secondary, Simle.*

*I wanted to carry on with my studies, but we didn’t have the money. Going to college was a dream, but we couldn’t afford to let me go and cover my brother’s schooling too. So, I put my studies on hold, and I started to save as I could. I can try my hand at anything, glass-cutting, furniture making, truck driving, soap selling. I got enough together to continue my studies, and complete class 12 in Management. I started thinking about the future.*

*I needed a job. Most of my friends in the village had gone abroad to the Gulf Countries. It’s difficult to get a good job here—and the money is better. I had a dream of a better life—so it didn’t take long to decide to leave for Saudi Arabia. The chance to gather new knowledge and experience of the world!*

*After I got to Riyadh, I quickly found a job as a waiter in a pizza company, working 9 hours a day and more. It wasn’t what I imagined: a new place, foreign language, different customs. I found it difficult to cope in an unfamiliar city—I felt lonely, restless, and sad. I missed home—my family, life in Thakre. The more anxious I got, the more I couldn’t sleep and didn’t want to talk to the people I worked with or make new friends. I began to feel worthless and depressed—why wasn’t I coping? I nearly gave it all up. But I knew I needed to send money home—and I owed money too, to the recruitment agency who had sent me here to Saudi Arabia. So, I had to keep going.*

*Luckily, I had a good friend and co-worker, Rajesh, who like me had come from Nepal. He noticed how sad and despondent I had become. I could talk to him. On a day off, we walked in a nearby park, and Rajesh shared his experiences with me: ‘Suman, I really know how you feel. I also struggled to adapt when I arrived here, and I confided in a close friend. He gave me some strategies to reduce my stress, and to take seriously the way I was feeling. It worked for me. Mental health is like physical health. Problems shouldn’t be played down but addressed with the same care and importance as a physical illness. If things don’t improve, it might be a good idea to seek additional support from healthcare professionals.*

*I listened to his advice and began to share how I was feeling. I tried out his techniques for coping better. Gradually I felt relief, somehow easier. When I missed my family in Nepal, I chatted with my parents and youngest brother on the phone. I listened to Nepali folk songs when I came home from work. I made friends, and we sang and danced when we gathered together. Instead of spending my time feeling sad and lonely, I decided to busy myself with learning Arabic, reading newspapers, and exploring the country with friends. I became used to the weather, local food and culture, and found out about the health services I could access.*

*During my time in the Gulf, I came down with a very high fever. I felt anxious without my family there. When the fever didn’t go down, I went to hospital for a check-up. The treatment was expensive, but the health Insurance I had from my employer made it easier to pay the bill. I was signed off for five days to rest and recover. I had time to read a novel, listen to songs, and write a poem – I often scribble them down to express how I’m feeling. After a few days I had recovered, and learned for myself from experience how important it is to keep mind and body healthy.*

*I started to think about how my patterns of behaviour made a difference to my feelings and began to identify what activities made me feel positive and happy. Each day I made sure they were part of my regular daily routine. I kept fit by doing light exercise as well as yoga and meditation. I made sure my food was nutritious, and my sleep routine was as regular as possible. I avoided using smoking and drinking to reduce my worries and anxieties—a quick fix which didn’t work and left me more worried about my physical health. Whenever I felt homesick, I calmly closed my eyes and visualised swimming in the Mahesh stream in my village or going to a religious fair in the Shiva Temple with my friends.*

*I returned to Nepal after two years. I got married, and opened a small restaurant in the village, to bring my dream of successful business home. These days the young men thinking of foreign employment come to me for advice, and I’m delighted to help them. I tell them: find out information about the world of work in your destination country before you go there—and think about how you are going to stay healthy abroad, in both mind and body. It will be much easier if you learn the language and are open to a different culture and customs.*

*Adversity teaches us valuable lessons. The pleasures and hardships in life are learned through experiencing the challenges of unfamiliar lands. It is down to you to take responsibility for your livelihood, lifestyle and actions. Everyone goes through stressful situations at some point. But we can build our resilience if we make sure that we have a support network of people around us, engage in activities we find personally meaningful and which make us happy, and take care of our physical health. That’s my message, which I learned from a friend—and I’m passing it on!*

### Phase 3: coproduction of the storytelling intervention

The coproduction workshops lasted approximately three hours each. In the first workshop, the group reflected on the findings from preceding phases. Drawing on these findings and the workers’ lived experiences, potential ideas for story plot and characters were discussed. During the discussion, several health and well-being issues were highlighted including the personal narratives of the migrant workers and their families. The second workshop focused on the story of psychosocial well-being, as prioritised by the migrant workers and their families, given the complexities of adversities faced abroad. This decision was based on evidence from previous phases and personal narratives of migrants and their families, as well as the importance of preventing distress and promoting psychosocial well-being of the migrant workers. The third workshop developed ideas for the structure, format and delivery of the intervention. The first draft of the story was created by the literary scholar with input from the coproduction group. The story was further developed through an iterative process (in-person and face-to-face conversations and meetings) and refined until agreement was reached. Overall, the participants expressed a positive experience of their involvement in the process, especially having a platform to share their stories, and how it shaped Suman’s story, the intervention. They reported that their perspectives and opinions were valued and listened to, and they seemed relaxed in the informal environment of the workshops.

Subsequently, academic collaborators with applied psychology backgrounds (DM and CB) were invited to comment on a draft of the story to ensure that the key components of psychological well-being (physical, economic, social, mental, emotional, cultural and spiritual determinants of health) were clearly framed in the story.^38^ Specific attention to the story development process enabled identification of features migrant workers and their families found appealing, especially focusing on the representation of migration, labour and health and well-being, as well as migrants’ agency and voice.

Once a story script had been agreed by all participants, a video was developed with the help of a professional videographer. Video was chosen as the preferred format instead of audio or print in both phase 1 (pilot survey) and by the coproduction group. The development of the video involved numerous iterative phases. The coproduction group was actively involved in all stages until the video was finalised (figure 1). The final story centres around the struggles of an archetypal male migrant worker and his coping strategies for dealing with adversities (box 1). An animated video with audio narration and subtitles can be accessed from the project website.^39^

## Discussion

To our knowledge, this is the first study to coproduce a public health intervention with Nepalese migrant workers. The developmental process was shaped by: our systematic review^4^; a formative mixed-method study that collected stakeholders’ perspectives on the health and well-being issues of Nepalese migrant workers^31^; narrative analysis of Nepalese literary resources; and a series of coproduction workshops involving migrant workers and their families. The systematic review identified mental health problems, occupational hazards, sexual health, healthcare access and infectious diseases as key health issues among migrant workers. The mixed methods study identified issues related to physical health (eg, pneumonia, kidney disease) as well as mental health (eg, anxiety, depression). Nepalese literary resources primarily portrayed separation, hopelessness and helplessness, and poor workplace environments. Based on these findings and working collaboratively with stakeholders, a culturally sensitive storytelling intervention was coproduced to support the psychosocial well-being of Nepalese migrant workers in GCC countries.

Coproduction is widely promoted as a method to enhance the utilisation of research and its impact. Nevertheless, it comes with its own challenges and there is no ‘one size fits all’ approach.^26 40^ As Hawkins *et al* highlight, coproduction is ‘both iterative and fluid’.^28^ The development of the storytelling intervention in our study was also a non-linear process, marked by numerous iterative phases and continual refinement prior to finalisation.^41^ It is important that coproduction incorporates inclusive and culturally sensitive approaches, taking into consideration stakeholders’ needs and capabilities to minimise resistance and distrust, and setting realistic expectations for those involved. We encountered resource and time constraints as key barriers in this process. Managing expectations and priorities for a diverse range of stakeholders was also sometimes challenging, and particularly during the development of story content. These issues have been also highlighted in previous studies involving coproduction.^40 42^

A particular strength of this study is the involvement of migrant workers, their families and other stakeholders throughout the life-span of the whole project. This participatory model of coproduction values the under-represented voice of migrants and their communities.^42^ Discussions held in the coproduction group suggested that the workshops fostered a welcoming environment where participants expressed a sense of unity and shared purpose. Similar to a previous study, participants in our project reflected that their motivation stemmed from the anticipation that their personal narratives had the potential to impact the health and well-being of migrant communities.^43^ Intervention development was additionally shaped by the rich cultural and historical contexts embedded in the Nepalese literature.

Stakeholders’ knowledge and acquisition of wider perspectives are paramount in generating high-quality evidence that is not only scientifically sound but also socially robust.^44^ The project connected the migrant workers with government policy-makers and various NGOs in Nepal. Individual and group meetings were held with migrant workers, representatives of Ministries of Health and Population and of Labour and Employment and the NGO representatives working in the field of migration. Involvement of these key stakeholders enabled us to develop an intervention based on the needs and priorities of stakeholders. The study involved multidisciplinary teams involving expertise in public health, psychology, demography, sociology, media and arts and humanities. Multidisciplinary collaboration is essential for contributing to the global effort on migration and for achieving evidence-based policy impacts.^45^

Labour migration is one of the key cultural narratives in contemporary Nepal. In addition to the activities outlined in the methods section, we organised two project symposia (one each in Nepal and UK) to raise awareness and create new approaches to understanding public health dimensions of migration. These activities were beneficial in cocreating knowledge and building a collaborative relationship, improving multidirectional communication of key issues related to migration. The Nepalese media play a significant role in shaping the country’s culture, society and politics. Hence, we published two newspaper articles on a Nepalese online news portal highlighting the health issues of migrant workers in GCC and the need for culturally appropriate interventions.

Reflecting cultural sensitivity in public health interventions is crucial to address the diverse need of the population and promoting equity. Culture is shaped by beliefs, values and practices that are socially established and shared by members of groups.^46^ Evidence suggests that interventions that consider cultural and linguistic diversity are not only more acceptable but also enhance engagement and compliance to the intervention. In terms of storytelling specifically, greater realism (ie, perceived authenticity or similarity to real life) has been found to increase narrative engagement, which is key to achieving transportation and identification that enable cognitive-affective changes linked to distal behavioural outcomes.^47^

For migrant populations, culturally sensitive interventions are not only beneficial but essential to ensure appropriate and effective care regardless of their cultural or linguistic background.^48^ Our study contributes to an emerging effort to develop culturally sensitive interventions with migrant workers.^6 49 50^ In practice, the cultural adaptation framework explained that it requires a dynamic, iterative approach to improve psychological well-being and also strengthen trust between migrants and healthcare systems. The services must use a patient-centred and culturally informed approach, understanding and respecting the cultural diversity of the migrant experience in mental healthcare.^51 52^ Previous studies have demonstrated that multimedia storytelling offers a promising approach for delivering culturally appropriate health education and may be particularly beneficial for promoting behavioural change in vulnerable populations, including those with low-health literacy.^53^,^55^ The coproduction approach in this study could provide a foundation and be adapted to develop public health interventions with other migrant groups, including marginalised and underserved communities.

There are some limitations to the overall project as well as specific to the coproduction phase. Phase 1 of the project, the systematic review, did not search grey literature and was limited to English language databases. Additionally, the site for the mixed-method study was purposively selected and covered only one rural municipality of one district. However, the demographics of those involved were similar to the wider population of Nepalese migrant workers.^9^ For phase 2, there were no central databases of Nepalese literature, and the search involved visiting libraries, academic institutions, approaching contemporary writers and consulting book publishers. Although this was a comprehensive approach, we may have missed some relevant literature. For coproduction (phase 3), it was challenging to find a meeting time that suited everyone, and some participants were unable to attend all sessions due to work and family commitments. Furthermore, informal discussions were held with the coproduction group regarding their experience in the coproduction process, but we were unable to gather formal feedback on the prototype due to COVID-19 restrictions imposed towards the end of the study (spring 2020). Despite these limitations, the study highlights the value of participatory methods in developing culturally sensitive public health interventions for marginalised groups and the utility of coproduced storytelling in migrant health contexts.

## Conclusion

This is the first study to develop a culturally appropriate storytelling intervention with Nepalese migrant workers and their families. The resulting intervention comprised an animated video with audio narration and subtitles, telling the story of an archetypal male migrant worker and their use of coping strategies for dealing with adversities. The coproduction of the video story built on scientific evidence pertaining to migrant health, themes from Nepalese literature and lived experience insights related to health and well-being issues in migrant contexts, especially in Gulf Countries. The project testifies to the value of participatory methods in the development of culturally sensitive public health interventions for marginalised groups, and points to the utility of coproduced storytelling formats in migrant health contexts in particular. Future research is needed to test the acceptability, feasibility and outcomes of this intervention, which has the potential to enhance the psychosocial well-being of migrant workers.

## Data Availability

All data relevant to the study are included in the article or uploaded as supplementary information.
