# Supplementary material for: Coproducing a culturally sensitive storytelling video intervention to improve psychosocial well-being: a multimethods participatory study with Nepalese migrant workers
Source: BMJ Open. 2025 Feb 17;15(2):e086280. doi: 10.1136/bmjopen-2024-086280 (PMC11836809; doi:10.1136/bmjopen-2024-086280)
Supplement: Supplementary file 1 [file bmjopen-2024-086280.reviewer_comments.pdf]

PEER REVIEW HISTORY

BMJ Open publishes all reviews undertaken for accepted manuscripts. Reviewers are asked to complete a checklist review form and are provided with free text boxes to elaborate on their assessment. These free text comments are reproduced below.

ARTICLE DETAILS

Title (Provisional)

Co-producing a culturally sensitive storytelling video intervention to improve psychosocial wellbeing: a multi-methods participatory study with Nepalese migrant workers

Authors

Paudyal, Priyamvada; Wasti, Sharada Prasad; Neupane, Pimala; Sapkota, Jib Lal; Watts, Carol; Kulasabanathan, Kavian; Silwal, Ram; Memon, Anjum; Shukla, Prajaya; Pathak, Ram Sharan; Michelson, Daniel; Berry, Clio; Moult, Alice; Simkhada, Padam; van Teijlingen, Edwin R; Cassell, Jackie

---

VERSION 1 - REVIEW

**Reviewer**	**1**
**Name**	**Ha, Toan**
**Affiliation**	**University of Connecticut**
**Date**	**24-Jul-2024**
**COI**	**No**

The authors conducted an interesting and innovative study using the co-production approach to develop public health interventions with migrant groups and the approach can be applied to larger audiences including marginalized community. I have some minor comments below

Introduction

Please start with a positive role of migrant workers, indicating that they contribute to the social and economic development. Then go on with their health risk outcomes.

Results

To gain a comprehensive understanding of migrant worker wellbeing, the study could benefit from including an assessment of the six core aspects of the Psychological Wellbeing (PWB) Scale in phase 1. These aspects, which measure overall wellbeing and happiness, are: Autonomy , Environmental Mastery ; Personal Growth ; Positive Relations With Others ; Purpose in Life ; and Self-acceptance.

To enhance clarity, it would be helpful to include a summary of the findings from the analysis of 49 relevant literary resources (conducted in phase 2) within Table 1.

For Phase 3, please describe in the process how participants and family members engage in the workshops, were they given any guidance to join the discussion,…?

**Reviewer**	**2**
**Name**	**Ibrahim, Mohd Ismail**
**Affiliation**	**Universiti Sains Malaysia, Department of Community Medicine**
**Date**	**21-Sep-2024**
**COI**	**No**

I reviewed the manuscript, and overall, it is well-written and presents an interesting project highlighting the need to improve the psychological wellbeing of a marginalized population.

The title of the study is generally appropriate and reflects the core essence of the research. However, it does not explicitly mention that the study employs a mixed-methods design, which is important for clarifying the research approach. A minor modification is suggested: "Co-producing a culturally sensitive storytelling video intervention to improve psychosocial wellbeing: a mixed-methods participatory study with Nepalese migrant workers."

The abstract provides important content that helps readers understand the study without needing to read the entire article. It gives a clear summary of the research. However, a few additions could make it more comprehensive. Specifically, including more details about the findings from each phase (systematic review, survey, and qualitative interviews) would strengthen the abstract's ability to fully inform the reader. Additionally, highlighting limitations such as the small sample size and geographic focus in the abstract would offer a more balanced overview of the study's scope.

The introduction effectively sets up the study, providing a strong rationale for the intervention and clearly identifying the gap it aims to address. The study objective is well-defined and constructed. However, the introduction would benefit from integrating a theoretical model or framework to offer a stronger conceptual foundation. A theoretical model could explain the mechanisms through which the storytelling intervention is expected to improve psychosocial wellbeing. It would also support the rationale for employing a culturally adaptive and participatory approach. Examples of proven theories include Social Cognitive Theory, Theory of Planned Behavior, and the Cultural Adaptation Framework.

The methodology is generally appropriate and robust for the study's objectives, but several areas could benefit from further elaboration to ensure methodological rigor and transparency:

• Mixed-Methods Design: The mixed-methods design is appropriate and aligns with the study's objectives. However, additional details regarding the systematic review process (e.g., search strategy, inclusion/exclusion criteria, and quality assessment of reviewed studies), survey design (e.g., sampling strategy, instrument development, and representativeness of the sample), and qualitative analysis (e.g., interview guides, coding process, and steps taken to ensure trustworthiness such as triangulation and member checking) would enhance the study's clarity and robustness.

• Data Collection: While the data collection methods are generally appropriate for the study's objectives, greater transparency regarding the sampling strategy, instrumentation, and data saturation would improve the methodological rigor.

• Statistical Analysis: Descriptive statistics used in the quantitative section are appropriate for the small sample size. However, adding inferential statistics where possible would enrich the analysis. For the qualitative data, the thematic analysis process, including coding procedures and inter-coder reliability, requires more detailed explanation.

• Data Integration: As a mixed-methods study, more explanation of how qualitative and quantitative data were integrated would be helpful. Clarifying whether a sequential or convergent design was used and how these data complemented each other would enhance the understanding of the study's overall findings.

The results are logically structured according to the three phases of the study, aligning with the mixed-methods design. However, the following areas could be improved:

• Themes and Subthemes: While the main themes are described, the subthemes are not adequately developed or structured. Adding a more detailed breakdown of subthemes would deepen the analysis and provide more insight into the data.

• Participant Quotes: The manuscript would benefit from the inclusion of direct quotes from participants to illustrate the findings, particularly in relation to the subthemes. This would add richness to the qualitative narrative and demonstrate a clear link between the data and the themes.

• Sociodemographic Details: The manuscript should include a detailed description of the sociodemographic characteristics of the participants to provide context for the heterogeneity of the findings and support generalizability.

The discussion effectively emphasizes the value of a culturally adaptive, co-produced storytelling intervention in addressing the psychosocial wellbeing of Nepalese migrant workers. However, several improvements could be made to strengthen the discussion:

1. Provide a more in-depth interpretation of the key findings from both the quantitative and qualitative components, explaining how these findings complement each other.

2. Explicitly link the findings to relevant theoretical frameworks.

3. Discuss how the cultural sensitivity of the storytelling intervention contributes to its potential success.

4. Elaborate on the potential policy implications of the findings, particularly in relation to migrant worker health, public health interventions, and culturally adaptive strategies.

The limitations of the study are discussed but could be expanded to improve transparency. It is important to address issues such as the small sample size and its impact on generalizability, the geographic limitation of focusing on only one district, potential selection bias, and the limitations of the co-production process.

VERSION 1 - AUTHOR RESPONSE

**Reviewers' comments**	**Our Response**
**Reviewer: 1**	
1.The authors conducted an interesting and innovative study using co-production approach to develop public health interventions with migrant groups and the approach can be applied to larger audiences eg marginalized community. some minor comments	Thank you very much for the positive feedback.
2.Introduction<br>Please start with a positive role of migrant workers, indicating that they contribute to the social and economic development. Then go on with their health risk outcomes.	Thank you. We have now added the information in the introduction section. Please see the first paragraph in the introduction section.
3.Results<br>To gain a comprehensive understanding of migrant worker wellbeing, the study could benefit from including an assessment of the six core aspects of the Psychological Wellbeing (PWB) Scale in phase 1. These aspects, which measure overall wellbeing and happiness, are: Autonomy , Environmental Mastery ; Personal Growth ; Positive Relations With Others ; Purpose in Life ; and Self-acceptance.	Thank you, our study focused on the co-development of the storytelling intervention with findings from phase 1 (systematic review and mixed-methods study) already published [1,2]. We appreciate your suggestion using aspects on PWB and we will certainly consider incorporating these in elements in assessment of psychological wellbeing in our future studies.<br>1. Health and well-being issues of Nepalese migrant workers in the Gulf Cooperation Council countries and Malaysia: a systematic review - PubMed

	2. Health and wellbeing of Nepalese migrant workers in Gulf Cooperation Council (GCC) countries: A mixed-methods study - PubMed
4.Results<br>To enhance clarity, it would be helpful to include a summary of the findings from the analysis of 49 relevant literary resources (conducted in phase 2) within Table 1.<br><br>For Phase 3, please describe in the process how participants and family members engage in the workshops, were they given any guidance to join the discussion,…?	As suggested, we have added the themes, key themes and example scripts from the literary resources relating to these in a table. Please see Table 1b in the revised manuscript.<br><br>We have now added this detail in the methods (please see Phase 3 on the section) and results (Phase 3 on the section)
**Reviewer: 2**<br>1. Overall, it is well-written and presents an interesting project highlighting the need to improve the psychological wellbeing of a marginalized population.	Thank you very much for the positive feedback.
2.The title of the study is generally appropriate and reflects the core essence of the research. However, it does not explicitly mention that the study employs a mixed-methods design, which is important for clarifying the research approach. A minor modification is suggested: "Co-producing a culturally sensitive storytelling video intervention to improve psychosocial wellbeing: a mixed-methods participatory study with Nepalese migrant workers."	We appreciate the suggestion. As number of methodological approaches were used (systematic review, pilot survey, qualitative interviews and participatory workshops), we believe that 'multi-method' is a more appropriate term than mixed-methods study. Hence, we have revised the title to: "Co-producing a culturally sensitive storytelling video intervention to improve psychosocial wellbeing: a multi-method participatory study with Nepalese migrant workers"
3.The abstract provides important content that helps readers understand the study without needing to read the entire article. It gives a clear summary of the research. However, a few additions could make it more comprehensive. Specifically, including more details about the findings from each phase (systematic review, survey, and qualitative interviews) would strengthen the abstract's ability to fully inform reader.<br><br>4.Additionally, highlighting limitations such as the small sample size and geographic focus in the abstract would offer a more balanced overview of the study's scope.	As mentioned above, we have already published the findings from the systematic review and the mixed-methods study (reviewer 1 response 4), hence we have provided only a summary of key findings in the abstract.<br><br>As suggested, the limitations have been added in the revised abstract.
5. The introduction effectively sets up the study, providing a strong rationale for the intervention and clearly identifying the gap it aims to address. The study objective is well-defined and constructed. However, the introduction would benefit from integrating a theoretical	Introduction (page 3 line 35-39) now provides theoretical context to storytelling interventions:

model or framework to offer a stronger conceptual foundation. A theoretical model could explain the mechanisms through which the storytelling intervention is expected to improve psychosocial wellbeing. It would also support the rationale for employing a culturally adaptive and participatory approach. Examples of proven theories include Social Cognitive Theory, Theory of Planned Behavior, and the Cultural Adaptation Framework.	Drawing on concepts from communication theory [5] and social cognitive theory [6], mechanistic descriptions of storytelling interventions have highlighted key processes including transportation (reducing cognitive resistance by encouraging the reader/listener to enter into the world of the narrator); and identification (enhancing the listener/viewer's acceptance of the values and beliefs portrayed in the story through narrative persuasion and modelling) [7].<br><br>[5] Fisher WR (1984). Narration as a human communication paradigm: The case of public moral argument. Communication Monographs, 52, 347–367.<br>[6] Bandura A (2002) Social cognitive theory in cultural context. Applied Psychol, 51(2), 269-290.<br>[7] Lee H. et al (2016). Storytelling/ narrative theory to address health communication with minority populations. Applied Nurs Res, 30, 58-60.
5. Methodology<br>The methodology is generally appropriate …but several areas could benefit from further elaboration to ensure methodological rigor and transparency:<br>• Mixed-Methods Design is appropriate and aligns with objectives. However, additional details regarding the systematic review process (e.g., search strategy, inclusion/exclusion criteria, quality assessment of studies), survey design (e.g., sampling strategy, instrument development, and representativeness of sample), and qualitative analysis (e.g., interview guides, coding process, and steps taken to ensure trustworthiness e.g. triangulation and member checking) would enhance clarity and robustness.<br>• Data Collection: .. greater transparency regarding the sampling strategy, instrumentation, and data saturation would improve the methodological rigor.<br>• Statistical Analysis: Descriptive statistics used are appropriate for the small sample size. However, adding inferential statistics where possible would enrich analysis. For the qualitative data, thematic analysis process, including coding procedures and inter-coder reliability, requires more detailed explanation.	 Thank you for your thoughtful and detailed feedback on the methodology. As mentioned previously, the findings from the systematic review and mixed-methods study have already been published [1,2]. These studies are open access and freely available to all readers. To avoid repetition of information, we have opted not to include the full methodology in this manuscript. Instead, we have cited these studies (citations 4 and 31 in the current manuscript) in the methodology section to guide readers to the relevant details for further reference.<br>1. Health and well-being issues of Nepalese migrant workers in the Gulf Cooperation Council countries and

• Data Integration: As a mixed-methods study, more explanation of how qualitative and quantitative data were integrated. Clarifying whether a sequential or convergent design was used and how these data complemented each other would enhance the understanding of the overall findings.<br>Results are logically structured according to three phases of the study, aligning with the mixed-methods design. However, following areas could be improved:<br>• Themes and Subthemes: …subthemes are not adequately developed or structured. Adding a more detailed breakdown of subthemes would deepen the analysis and provide more insight into the data.<br>• Participant Quotes: would benefit from inclusion of direct quotes from participants to illustrate findings, particularly in relation to subthemes. This would add richness to qualitative narrative and demonstrate a clear link between data and the themes.<br>• Sociodemographic Details: include a detailed sociodemographic characteristic of participants to provide context for heterogeneity of findings and support generalizability.	Malaysia: a systematic review - PubMed<br>2. Health and wellbeing of Nepalese migrant workers in Gulf Cooperation Council (GCC) countries: A mixed-methods study - PubMed
7. Discussion effectively emphasizes value of a culturally adaptive, co-produced storytelling intervention in addressing the psychosocial wellbeing of Nepalese migrant workers. Several improvements could strengthen the discussion:<br><br>a. Provide a more in-depth interpretation of the key findings from both the quantitative and qualitative components, explaining how these findings complement each other.<br><br>b. Explicitly link the findings to relevant theoretical frameworks.	a) As stated earlier, mixed-methods study has been published. Health and wellbeing of Nepalese migrant workers in Gulf Cooperation Council (GCC) countries: A mixed-methods study - PubMed<br><br>b) Thank you, on p 11 line11-26, we have added the following to link Results/Discussion with theoretical concepts now referenced in the Introduction (see response to Comment 5 above):<br><br>In terms of storytelling specifically, greater realism (i.e., perceived authenticity or similarity to real life) has been found to increase narrative engagement, which is key to achieving transportation and identification that enable cognitive-affective changes

c. Discuss how cultural sensitivity of storytelling intervention contributes to its potential success. Elaborate on potential policy implications, particularly in relation to migrant worker health, public health interventions, and culturally adaptive strategies.<br><br>d. Limitations could be expanded to improve transparency. Address issues such as the small sample size and its impact on generalizability, the geographic limitation of focusing on only one district, potential selection bias, and limitations of co-production process.	linked to distal behavioural outcomes [9]<br><br>[9] Busselle, R., & Bilandzic, H. (2009). Measuring narrative engagement. Media psychology, 12(4), 321-347.<br><br>c. Thank you; we have now revised and include these in Discussion.<br><br>d. We have added the limitations of the study in the revised texts. Please see page 11, line 34-41.

VERSION 2 - REVIEW

Reviewer 2

Name Ibrahim, Mohd Ismail

Affiliation Universiti Sains Malaysia, Department of Community Medicine

Date 06-Dec-2024

COI

I have read the revised version of the manuscript, and thank you for your effort to address the reviewers' concerns. To further enhance the clarity and transparency of the manuscript, I suggest including a statement in the introduction or methodology explicitly noting that this study is part of a larger project with multiple objectives. This would provide readers with a clearer understanding of the broader research context and justify the citations of previously published findings. Additionally, to improve the flow of the methodology and findings, I recommend summarizing the key results of the two prior publications (systematic review and mixed-methods study) in the revised manuscript. This summary could succinctly highlight the main findings of these earlier phases, establishing a coherent narrative that connects the previous work with the development and implementation of the storytelling intervention presented in this paper. Such an approach will address concerns about self-citation while enhancing the overall readability and comprehension of the study.

VERSION 2 - AUTHOR RESPONSE

Comments	Response
**Reviewer: 2**<br><br>1. I have read the revised version of the manuscript and thank you for your effort to address the reviewers' concerns. To further enhance the clarity and transparency of the manuscript, I suggest including a statement in the introduction or methodology explicitly noting that this study is part of a larger project with multiple objectives. This would provide readers with a clearer understanding of the broader research context and justify the citations of previously published findings.	 <br>1.Thank you for the suggestion; we have now included this information in the first paragraph of the methodology section (page 4).
2. Additionally, to improve the flow of the methodology and findings, I recommend summarizing the key results of the two prior publications (systematic review and mixed-methods study) in the revised manuscript. This summary could succinctly highlight the main findings of these earlier phases, establishing a coherent narrative that connects the previous work with the development and implementation of the storytelling intervention presented in this paper. Such an approach will address concerns about self-citation while enhancing the overall readability and comprehension of the study.	2. Again, thank you for the suggestions. As advised, we have expanded the results section incorporating the findings from the previous two publications. Please see Phase 1 on the results section (page 6).
